# Grossesse ovarienne: à propos de 3 cas et une revue de la literature

**DOI:** 10.11604/pamj.2016.25.128.10834

**Published:** 2016-11-02

**Authors:** Haingo Voahangy Rabetafika Ranaivoson, Volahasina Françine Ranaivomanana, Lalaina Nomenjanahary, Tsitohery Francine Andriamampionona, Nantenaina Soa Randrianjafisamindrakotroka

**Affiliations:** 1 UPFR Anatomie et Cytologie pathologiques du CHU/JRA, Antananarivo, Madagascar; 2 UPFR Anatomie et Cytologie pathologiques du CHU Anosiala, Madagascar; 3 UPFR Anatomie et Cytologie pathologiques du CHU Andrainjato Fianarantsoa Madagascar

**Keywords:** Grossesse ovarienne, facteurs de risques, diagnostic, histologie, Ovarian pregnancy, risk factors, diagnosis, histology

## Abstract

La grossesse ovarienne représente une entité rare parmi les grossesses ectopiques où l'ovaire est le siège de la nidation. Son diagnostic nécessite une démarche bien codifiée. Les particularités des facteurs déterminant, des caractéristiques histopathologiques et évolutives des grossesses ovariennes nous a conduit à porter un intérêt pour cette forme de grossesse ectopique. Nous rapportons 3 cas de grossesse ovarienne diagnostiqués dans notre service. Les trois femmes ont entre 30 à 42 ans et un terme de 13 à 37 semaines d'aménorrhée. Elles sont toutes révélées par des douleurs abdominales d'intensité variable avec un état de choc. L'examen anatomopathologique de l'annexe droite, siège caractéristique des grossesses ovariennes, a permis de confirmer le diagnostic. Elles sont toutes des grossesses ovariennes primitives juxta-corticales. La grossesse ovarienne est une entité très rare de la grossesse extra-utérine qui présente certaines particularités. Son diagnostic est difficile et se base sur des constations per-opératoires. La présence de la zone de nidation ovarienne à l'examen histopathologique est optimale pour confirmer le diagnostic. Actuellement, on admet que la grossesse ovarienne est la forme de grossesse ectopique qui peut aller à terme ou même jusqu'à une naissance vivante.

## Abstract (en)

Ovarian pregnancy is a rare event among women with ectopic pregnancy in whom the ovary is the site of implantation. Its diagnosis requires a well codified approach. The peculiarities of the determining factors, of the histopathological and evolutionary features typical of ovarian pregnancies led us to focus on this form of ectopic pregnancy. We report 3 cases of ovarian pregnancy diagnosed in our Department. The three women were between 30 and 42 years, at 13 to 37 weeks of amenorrhea. All women had abdominal pain of varying intensity associated with shock. Anatomopathological examination of the right annex, normal site of implantation of ovarian pregnancies, confirmed the diagnosis. All women had juxtaposition ovarian cortical pregnancy. Ovarian pregnancy is a rare event among women with ectopic pregnancy, having specific peculiarities. Its diagnosis is difficult and is based on preoperative findings. The identification of the ovarian implantation on histopathological examination is ideal for confirming the diagnosis. Currently, it is assumed that ovarian pregnancy is the form of ectopic pregnancy which can develop to term or even result in a live birth.

## Introduction

La grossesse ovarienne (GO) est une variété de grossesse où l'ovaire est le siège de la nidation [1]. Elle occupe une place particulière parmi les grossesses ectopiques en raison de sa rareté qui est liée d'une part à sa définition qui prend en compte des critères anatomiques, et d'autre part à des démarches diagnostiques bien codifiées. Contrairement, aux autres types de grossesse extra-utérine (GEU) la GO reste un phénomène isolé et exceptionnel, indépendant des facteurs de risques habituels. D'autant plus que le mécanisme exact aboutissant à une GO est encore mal élucidé. Par rapport aux autres GEU, d'autres formes de révélation de la GO ont été rapportées comme quoi la GO peut évoluer jusqu'au 2^ème^ trimestre, voire jusqu'à terme [2]. Les objectifs de ce travail consistent à analyser les facteurs déterminants de la GO, d'étayer les particularités étiopathogéniques, histopathologiques et évolutives de cette grossesse ectopique.

## Patient et observation

### Observation n°1

**Figure 1:**
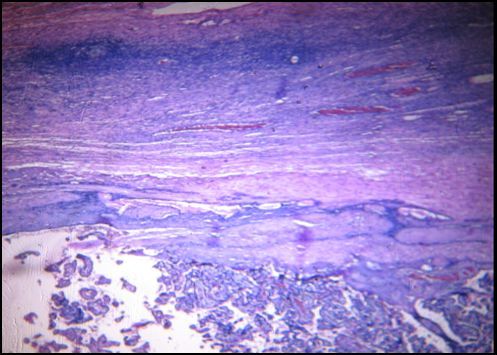
Cas n°1: grossesse ovarienne juxta-corticale. Hématéine et eosine x100

Madame R… Nathalie âgée de 31ans, G2 P1 A0, a été admise dans le service de Maternité Befelatanana pour un état de choc associé à une douleur abdominale aigüe et une hémorragie génitale abondante. Aucune notion de prise de contraception n'a été révélée. Elle a eu ses dernières règles 8 mois auparavant de son admission. L'échographie pelvienne a montré une grossesse extra-utérine sans Activité Fœtale (ACF) estimée à 36 Semaine d'Aménorrhée (SA) 2 jours. Une laparotomie d'urgence a été décidée qui a permis l'extraction d'un fœtus sans vie situé en dehors de l'utérus et une annexectomie droite. L'aspect macroscopique des prélèvements adressés dans notre service a montré un placenta de 27x34x4cm avec un cordon ombilical de 10x5cm, et une trompe de 7x15cm. Les tranches de section réalisées sur le placenta montrent une plage jaunâtre de 2x1, 5x1cm avec des cavités kystiques de 0,2 à 0,4cm contenant du liquide gélatineux correspondant à l'ovaire. A l'examen histologique, on observe au sein du parenchyme ovarien dans la région corticale, un enduit de fibrine avec des cellules trophoblastiques migratrices et des villosités choriales matures (Figure 1). En conclusion, il s'agit d'une grossesse ovarienne estimée à 36-37SA.

### Observation n°2

**Figure 2:**
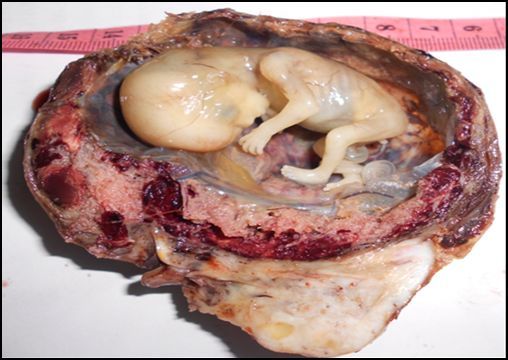
Cas n°2: aspect macroscopique après ouverture

**Figure 3:**
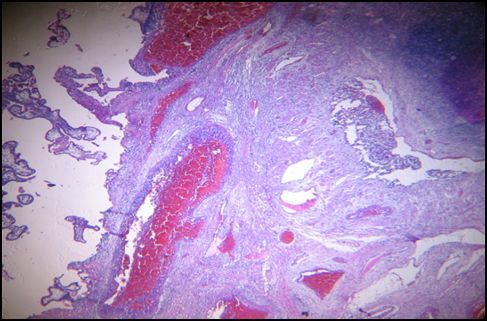
Cas n°2: zone de nidation. Hématéine et eosine x40

Madame R… Nicole âgée de 33ans, G3 P2 AO, a été admise dans le Service des urgences de l'HU/JRA pour un état de choc associé à une douleur abdominale aigüe, une hémorragie génitale abondante. Ses dernières règles remontent 3 mois auparavant de son admission. Elle a présenté dans ses antécédents une opération césarienne et a utilisé une méthode contraceptive, type Lévonogestrel* jusqu'au jour de son admission. L'échographie abdomino-pelvienne a montré une masse arrondie en regard de l'annexe droite associée à une hémopéritoine abondante. Une laparatomie en urgence a été effectué qui a permis l'exérèse de la masse et une annexectomie droite. L'aspect macroscopique des prélèvements adressés a montré un ovaire de 9x9x5cm, muni d'un sac gestationnel de 7 cm de grand axe. A la coupe, le sac gestationnel laisse voir un liquide citrin et un fœtus de sexe masculin (Figure 2). A l'examen histologique, on observe dans la partie corticale du parenchyme ovarien, des caillots sanguins enserrant quelques villosités choriales normalement développées pourvues de fentes vasculaires avec des globules rouges, associées à quelques cellules trophoblastiques migratrices (Figure 3). En conclusion, il s'agit d'une grossesse ovarienne estimée à 13 SA.

### Observation n°3

**Figure 4:**
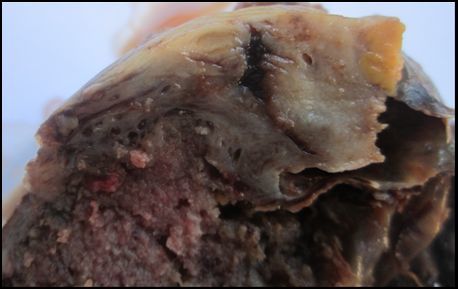
Cas n°3: aspect macroscopique après ouverture

**Figure 5:**
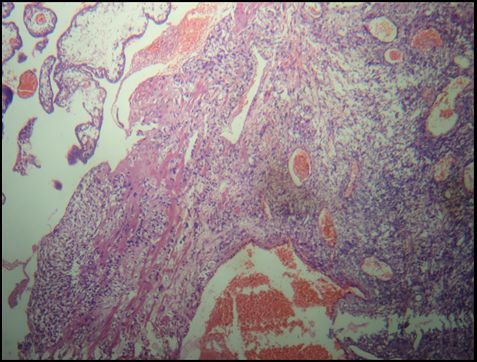
Cas n°3: zone de nidation. Hématéine et eosine x40

Madame B… âgée de 42ans, G8 P7 A0. Dans ses antécédents, aucune notion de prise de contraception. La date de ses dernières règles est inconnue. Elle a été admise à la maternité de Befelatanana pour un état de choc associé à une douleur abdominale et une hémorragie génitale abondante. Devant cet état une laporotomie d'urgence a été effectuée associant l'extraction d'une masse rompue aux dépens de l'annexe droite et une annexectomie droite. L'examen macroscopique du prélèvement adressé dans notre service dépourvu de fœtus, montre à la coupe, un sac gestationnel avec un placenta de 3x2x2,5cm sur lequel s'insère un cordon ombilical de couleur blanchâtre de 2,5x1,5x1cm. La zone d'insertion placentaire est accolée à ovaire de 1,5x1x0, 5 cm (Figure 4). A l'examen histologique, on observe dans le parenchyme ovarien un corps jaune gravidique et un enduit de fibrine avec des cellules trophoblastiques correspondant à la zone de nidation. Cette dernière est bordée par des villosités choriales de différentes tailles dont les vaisseaux sanguins contiennent des globules rouges (Figure 5). En conclusion, il s'agit d'une grossesse ovarienne estimée à 17SA.

## Discussion

Les GO représentent les premiers sites de localisation de GEU rares. La grossesse ovarienne a été suspectée pour la première fois par Mercureus en 1614 et prouvée à partir d'autres travaux cités par Grall [3]. Leur fréquence est estimée à 2-3% des GEU, ce qui représente une incidence d'environ 1/2500 à 1/5000 naissances [4]. Ces fréquences sont celles généralement annoncées dans la littérature [5, 6]. Dans notre étude, elle représente 4,65% des grossesses extra-utérines diagnostiquées dans notre Service. D'autres auteurs comme Sergent et Seinera, par contre estiment que la GO est beaucoup plus fréquente qu'on ne le pense [7, 8]. Par exemple Sergent [8], a retrouvé que l'incidence est évaluée à 1 pour 1400 naissances dans son étude. En définitif, les fréquences avancées sont discordantes. Cette disparité s'explique selon Grall [3] au manque de rigueur dans le respect des critères anatomiques et histologiques intervenant dans le diagnostic des grossesses ovariennes.

La population à risque est un peu différent de celles des patientes présentant une GEU tubaire puisqu'elle est représentée par des femmes jeunes, le plus souvent fertiles, multipares et portant un DIU [7]. Dans leur série, Riethmeller et al. ont trouvé deux cas de GO chez des femmes plus âgées, infertiles et sans DIU [9]. La fertilité pour nos 3 cas, et la multiparité pour un de nos cas sont les facteurs de risque recensés dans notre étude. L'âge de survenu de GO de nos 2 cas est proche de celle décrite dans une étude effectuée en Côte d'Ivoire [10] qui est comprise entre 20 et 34 ans. Pour E. Philippe [11], l'âge moyen d'apparition des GO est de 29 ans. Selon le nombre de parité, deux de nos cas ont une parité moyenne, tandis qu'une de nos patientes est multipare. Les avis des auteurs sont partagés sur ce facteur favorisant dans la genèse de GO: d'après Grall [3], la parité ne semble pas jouer un rôle car sur ses 4 cas, il a relevé 2 cas chez des secondigestes et 2 cas chez des multigestes soit 50%; pour sa part, Philippe [11] affirme que les multipares sont porteuses de GO dans 73 à 84% des cas.

Les variations de résultat sur le rôle de la parité dans la genèse de GO, exprime leur corrélation, mais cela est encore mal établi. La symptomatologie clinique est sans grande particularité, les douleurs abdominales, les retards de règles et les métrorragies sont les plus souvent présentés [5, 12]. Les douleurs correspondent à la rupture de la capsule ovarienne par la GO et à la constitution de l'hémopéritoine [8, 13]. Les patientes sont le plus souvent vues dans un contexte d'urgence, avec hémopéritoine important voire même en état de choc hypovolémique [13], ce sont des signes rapportés dans nos 3 cas. Mais d'autres circonstances ont été rapportées. Comme celui de Pan et al. un cas original de GO dans un tableau clinique de torsion annexielle a été rapporté [14]. De même, de très rares observations après hystérectomie inter-annexielle ont été rapportées [5, 11].

L'examen anatomo-pathologique revêt une importance capitale car c'est lui qui permet de confirmer le diagnostic de GO. Il a pour but d'éliminer les grossesses abdominales primitives, celles greffées sur l'ovaire mais provenant d'un avortement tubo-abdominal, et celles où l'ovaire n'est pas le siège exclusif de la nidation, d'après les critères anatomiques de Spielberg en 1878 [3] :la trompe du côté atteint, y compris le pavillon, doit être indemne de toute lésion ; le sac ovulaire doit occuper la place anatomique habituelle de l'ovaire; l'ovaire et le sac gestationnel doivent être reliés à l'utérus par le ligament utéro-ovarien ; il doit exister du tissu ovarien au sein du sac ovulaire [ce qui sous-entend la confirmation histologique de la présence de villosités choriales au sein du tissu ovarien]. A partir des critères anatomiques définis par Spielberg et Riethmiller [9], plusieurs classifications de GO ont été proposées par Baden [15], et Philippe [11] (Tableau 1). Dès lors, sur le plan histologique, des villosités choriales doivent être retrouvés au sein du tissu ovarien [9]. Si l'on se réfère à la définition décrit par Spielberg [9], seul le respect des 4 critères apporte le diagnostic de certitude des GO. Aucune de nos patientes ne satisfait complètement aux critères de cette définition. Pour autant, les aspects macroscopiques et histologiques sont en faveur d'un diagnostic de GO. Sergent et al. [8] rappellent que les critères cités ci-dessus n'intègrent pas les méthodes modernes de diagnostic, de traitement et de suivi des GEU. Ils paraissent insuffisants voire inadaptés, constituants un biais et entraînant une probable sous-estimation du taux de GO. Cette équipe propose donc d'associer les 4 critères de Spielberg à: 1) l'existence d'une GEU affirmée par un taux d'HCG plasmatique supérieur à 1000UI /L associé à une vacuité utérine en échographie endovaginale, la fausse couche spontanée étant exclue par l'absence ou le faible volume des métrorragies; 2) l'atteinte ovarienne confirmée par l'exploration chirurgicale, avec saignement ou visualisation de trophoblaste à son niveau, voire présence d'une formation kystique ovarienne atypique; 3) la présence de trompes saines; 4) la décroissance et négativation des taux de HCG plasmatiques après traitement de l'ovaire.

**Tableau 1:** Les principales classifications de grossesse ovarienne

AUTEURS	GROSSESSE OVARIENNE PRIMITIVE	GROSSESSE OVARIENNE SECONDAIRE
BADEN [19]	Grossesse primitive intra folliculaire et extra folliculaire	Grossesse combinée
PHILIPPE [14]	Grossesse intra folliculaire, Grossesse juxta-folliculaire, Grossesse juxtacorticale, Grossesse interstitielle	Grossesse corticale

En outre, si l'histologie retrouve du matériel ovulaire, même si celui-ci n'est pas entouré de tissu ovarien, le diagnostic de GO pourra être retenu dans la mesure où le prélèvement préopératoire intéresse l'ovaire. Ce qui est valable pour nos 3 cas, où on a la pièce d'annexectomie avec le matériel ovulaire. En plus, on a bien retrouvée la zone de nidation dans le parenchyme ovarien qui correspond à un enduit de fibrine avec des cellules trophoblastiques migratrices.

Selon le siège de la GO, nos 3 cas se développent aux dépens de l'ovaire droit. La GO selon Spiegelberg [16] est caractérisée par sa survenue habituelle dans le côté droit, du fait que: 1) la dimension normale de l'ovaire droit (16mm x19mm) est largement inférieure à celle de l'ovaire gauche (35mm x18mm); 2) une partie du parenchyme de l'ovaire droit se transforme souvent en une cavité kystique; 3) la paroi de cette cavité et l'ovaire ont la même structure histologique, dans cette cavité on retrouve habituellement les restes de fœtus et le vestige placentaire.

L'étiopathogénie des GO n'a pas été clairement définie. Plusieurs hypothèses s'opposent mais le mécanisme semble être celui d'un reflux transtubaire de l'ovocyte fécondé vers l'ovaire. Novak [17] rappelle les trois théories principales avancées pour expliquer la pathogénie, dont deux pour la GO primitive: 1) primo, la théorie d'une fécondation intra- folliculaire où un ovule non expulsé est fécondé à l'intérieur du follicule non rompu, par le spermatozoïde. Cette théorie est probablement erronée puisqu'on sait que l'ovocyte, pour être fécondable, doit subir une maturation nucléaire et cytoplasmique. Ces phénomènes doivent avoir lieu hors du follicule ; 2) secundo, la théorie de fécondation extra-folliculaire de Baden et Heins [15] : la fécondation se fait en dehors du follicule et la nidation est ovarienne, l'œuf s'implante préférentiellement sur la cicatrice de l'ostium folliculaire d'origine riche en fibrine et en néocapillaires [7]. D'un point de vue histologique, cette implantation correspond aux formes intrafolliculaires et juxtafolliculaires [18]. Plus rarement, l'implantation va se faire plus à distance du corps jaune, voire sur l'ovaire controlatéral correspondant aux formes juxtacorticales et interstitielles. Cette deuxième théorie sur l'implantation de l'œuf sur la partie corticale de l'ovaire explique la pathogénie de nos 3 cas ; 3) tertio, la théorie d'une greffe ovarienne d'une GEU issue d'un avortement tubo-abdominal [19].

D'autre part, Philippe E. [20] a envisagé le rôle possible de l'endométriose dans l'étiologie des GO. Quel que soit d'ailleurs le mécanisme en cause, il est probable que celui-ci n'est pas unique dans tous les cas, ce qui explique les différents types de GO observées.

Selon le terme, la plupart des GO sont abortives, d'ailleurs c'est ce que rapportent beaucoup d'auteurs où la GO est diagnostiquée avant les 12 SA [8]. Certaines GO peuvent évoluer jusqu'au 2^ème^ trimestre [5] voire d'avantage. Shahabuddin et Chowdhury [2] ont eu un cas de GO dans le cadre d'une grossesse hétérétopique, allant jusqu'à terme. Et pour terminer, d'après une étude en 1983 sur les 306 GO, 22 aboutissent à la naissance d'enfants survivants [5].

## Conclusion

Notre étude fait apparaître malgré un échantillon très réduit, qu'il existe à Madagascar des cas de GO. La particularité de notre étude réside sur le fait que, les GO rapportées présentent quelques aspects particuliers : elle survient parfois à un âge avancé, sont diagnostiqués dans le deuxième et troisième trimestre de la gestation, et sont toutes des GO primitives juxta-corticales.
